# What’s in a name? A preliminary event-related potential study of response to name in preschool children with and without autism spectrum disorder

**DOI:** 10.1371/journal.pone.0216051

**Published:** 2019-05-07

**Authors:** Rebecca P. Thomas, Leah A. L. Wang, Whitney Guthrie, Meredith Cola, Joseph P. McCleery, Juhi Pandey, Robert T. Schultz, Judith S. Miller

**Affiliations:** 1 Department of Psychological Sciences, University of Connecticut, Storrs, Connecticut, United States of America; 2 Center for Autism Research, The Children’s Hospital of Philadelphia, Philadelphia, Pennsylvania, United States of America; 3 Department of Psychology, University of Pennsylvania, Philadelphia, Pennsylvania, United States of America; 4 Department of Psychology & Kinney Center for Autism Education and Support, Saint Joseph’s University, Philadelphia, Pennsylvania, United States of America; 5 Perelman School of Medicine, University of Pennsylvania, Philadelphia, Pennsylvania, United States of America; Massachusetts General Hospital, UNITED STATES

## Abstract

The ability to selectively respond to one’s own name is important for social and language development, and is disrupted in atypically developing populations (e.g., autism spectrum disorder). Research with typically developing samples using event-related potentials (ERPs) has demonstrated that the subject’s own name (SON) is differentiated from other stimuli at both early sensory and later cognitive stages of auditory processing. While neural indices of response to name have been researched extensively in adults, no such studies have been conducted with typically developing preschool children or children with autism spectrum disorder (ASD). The present study investigated ERP response to name in a sample of typically developing (TD) preschoolers (*n* = 19; mean age = 4.3 years) as well as a small, exploratory comparison group of preschoolers with ASD (*n* = 13; mean age = 4.4 years). TD preschoolers exhibited significantly greater negativity to SON over frontal regions than to an unfamiliar nonsense name, consistent with the adult SON negativity component. This component was present whether the name was spoken by a parent or an unfamiliar adult, suggesting that it reflects SON-specific processing rather than broad self-relevant information processing. Comparing preschoolers with ASD to the TD children revealed a significant SON negativity component across both groups. The amplitude of the SON negativity response was significantly correlated with social variables in the ASD group, though these correlations did not survive correction for multiple comparisons. This study is the first to demonstrate the presence of the SON component in preschool children with and without ASD.

## Introduction

Names are some of the most influential auditory stimuli in a child’s environment. Studies have suggested that an infant’s own name is an early social cue that guides their attention to meaningful stimuli in the environment and facilitates skills such as social referencing [1]. Evidence indicates that typically developing infants orient to the sound of their own names by the time they are six months old [2,3], and that they use familiar names to segment speech in early language comprehension [4]. Names remain one of the most socially important stimuli as children experience an incredibly rapid expansion of social, communicative, and language skills from infancy to early childhood.

Previous research into neural responses to name using event-related potentials (ERPs) has provided further evidence of the unique salience of a person’s own name. ERPs reflect changes in electrical activity in the brain in response to a stimulus, allowing researchers to measure stimulus processing even in the absence of an observable behavioral change (such as turning your head toward someone calling your name). Studies examining ERP response to hearing one’s name demonstrate its differentiation from other stimuli at both early sensory and later cognitive stages of processing.

In early sensory processing, a greater N100 response to the subject’s own name (SON) versus an unfamiliar name has been reported for both adults [5,6] and school-aged children [7], although this may reflect detection of a familiar name rather than SON specifically [6]. Multiple studies have identified another early ERP response, believed to be an index of pre-attentive auditory detection, called SON negativity [8–10]. The SON negativity component consists of a negative deflection following the P200 response that is greater to the SON than to other highly familiar and unfamiliar names, and which is independent of the frequency with which the SON is presented [9,10].

In later stages of cognitive processing, hearing one’s own name produces enhanced responses in components associated with attentional orienting and cognitive appraisal of auditory stimuli. For example, the frontocentral P300 is an index of attentional orienting to a stimulus and occurs in response to contextually significant stimuli [10,11]. A number of studies have demonstrated robust P300 responses to SON [10,12–16]. The P300 to SON occurs even when it is not explicitly task-relevant, such as in passive oddball paradigms [10,12–15]. It has also been found to persist during sleep [16] and in other minimally conscious states [17]. However, this response is context-dependent as evidence suggests that it is only elicited when the SON is presented infrequently relative to the other stimuli [10].

In addition to P300 responses, sustained positivity over parietal regions during later stages of processing (i.e., 400 milliseconds or more post-stimulus onset) has been observed across several different experimental paradigms, including those with equiprobable presentation of SON and other auditory stimuli [6,8,14,18]. Such increased late parietal positivity (LPP) is thought to reflect deeper cognitive processing of self-relevant stimuli [8]. Greater LPP occurs in response to hearing SON spoken by a familiar voice versus an unfamiliar voice [14] and to hearing SON versus other familiar and unfamiliar names [6,8,18]. Taken together, evidence of enhanced P300 and LPP responses to SON demonstrate that one’s own name is a salient social stimulus that elicits specific higherlevel cognitive processing mechanisms.

One possible interpretation of these findings is that these components index processing of self-relevant stimuli, of which SON is a particularly strong example. Another explanation is that these components reflect the involvement of SON-specific processes in the brain. Several studies have included both familiar and unfamiliar other names as control stimuli [6,10,19], or have compared response to SON spoken by a familiar voice to SON spoken by an unfamiliar voice [14]. However, no ERP studies have included a fully crossed 2x2 design in which a familiar voice and unfamiliar voice each say both the SON and an unfamiliar name. In the present study we utilize a fully crossed design to determine whether the components found to index response to name reflect self-relevant information processing or own-name processing more specifically.

In addition, despite the clear importance of selectively responding to one’s own name during childhood, very little research has been conducted on the neural basis of response to name in children. To our knowledge, there are only three studies which have investigated the ERP response to SON in infants and children. Parise and colleagues found that infants differentiate their own name from a stranger’s name by five months of age [1]. However, the pattern of the infants’ neural response to their own name was substantially different from that of typical adults. This may suggest that the mechanisms underlying response to name are still immature in young infants. Key and colleagues demonstrated that children 4–12 years of age exhibited increased P300 responses to both SON and the name of a familiar other in a passive oddball paradigm [19]. Finally, Bathelt et al. examined ERP response to SON and an unfamiliar name in eight- to twelve-year-old typically developing children and children with congenital visual disorders [7]. Results indicated an enhanced N100 response to SON in both groups and a SON negativity component in the typically developing children that was attenuated over mid-central and right-frontal regions in the visually impaired group. Thus, current evidence indicates that several adult ERP indices of response to SON, including enhanced N100, P300, and SON negativity, are present in typically developing school-aged children, but not in young infants.

Despite these data, it remains unclear at what point in development these mature SON components emerge. Given the rapid growth in social and language skills that occurs during the preschool years, it is possible that the mechanisms underlying response to SON also mature during this time. However, none of the existing studies examining the neural response to SON in children included participants between one and four years of age. Furthermore, the sole study to include preschool children [19] incorporated a broad age range (four to twelve years) and used an oddball paradigm to examine only the P300 component. As such, the nature of the context-independent brain response to SON in younger children remains uncertain and unclear.

A better understanding of the neural mechanisms underlying response to name in young children has implications for atypical development as well. Response to name is a widely studied topic within the autism spectrum disorder (ASD) literature. Prior research has measured behavioral responses to naturally occurring social stimuli and found that children with or at-risk for an ASD diagnosis were more likely to fail to orient to a range of social stimuli, including the sound of their own name [20–23]. In fact, a reduced rate of behaviorally responding to one’s own name has recently been shown to be an early, specific indicator of ASD [24]. Despite the clinical importance of reduced behavioral response to name in ASD, relatively little is known about the neural mechanisms underlying this diminished response.

Although some previous ERP research has been conducted on response to name in adults with ASD, no study to date has examined which ERP components are associated with response to name in children with ASD. One ERP study in adults with ASD reported differences in late cognitive appraisal of own name [6]. Source localization indicated that these differences were linked to activity in the right temporo-parietal junction, an area which other evidence has suggested is heavily involved in social processing and theory of mind [25–27]. However, it is unclear when these neural processing differences develop and whether they are present in early childhood. Furthermore, while previous literature has examined the effect of familiarity of voice in typically developing adults [14], how these social-contextual factors influence cognitive processing of SON in individuals with ASD remains unknown.

To address these gaps in the literature, we present two experiments investigating brain response to name in preschoolers. In Experiment 1, we use naturalistic social stimuli in an ERP study with typically developing preschool children to examine how neural responses to name are influenced by speaker familiarity (familiar vs. unfamiliar) and stimulus type (own vs. nonsense name). In order to shed light on the mechanisms underlying response to name in atypical development, Experiment 2 presents preliminary data regarding whether there are group differences in neural response to own name in preschool children with ASD relative to typically developing children.

## Materials and methods

### Participants

The study sample consisted of 59 three- to five-year-old children who participated in a larger study of early development in ASD and developmental disabilities. The study recruited both typically developing (TD) children (*n* = 30) and children with a diagnosis of ASD (*n* = 29) (19 TD and 13 ASD with usable data; see “Analysis of ERP data” below). Participants were recruited through multiple sources, including online advertisements on the webpage for the Center for Autism Research (CAR), flyers posted on a recruitment board at The Children’s Hospital of Philadelphia (CHOP), and recruitment emails sent to families who agreed to be contacted about participating in research at CAR. Children were excluded from participation if they were born premature, had a history of traumatic brain injury or other significant medical or neurological abnormalities, an uncorrectable visual disorder, profound intellectual disability or sensorimotor impairment that precluded valid use of diagnostic instruments, or if English was not their primary language. TD participants were excluded if they experienced delays suggestive of autism-like impairment, a family history of ASD, or a score above 11 on the Social Communication Questionnaire (indicative of autism risk [28]).

All participants received a developmental assessment (Mullen Scales of Early Learning [29]) conducted by a PhD- or Masters-level clinician supervised by a licensed clinical psychologist or neuropsychologist. Parents were asked to complete several measures of their child’s social, adaptive, and language skills, including the Social Responsiveness Scale (SRS-2 [30]; N: 18 TD, 13 ASD) and the Vineland Adaptive Behavior Scales (Vineland-II [31]; N: 17 TD, 11 ASD). The Vineland-II provides a broadband measure of adaptive behaviors relevant to everyday functioning, and is valid across a range of developmental levels. Participants younger than 4 years of age received the SRS-2 Preschool Form (N: 9 TD, 5 ASD), while those 4 years of age or older received the SRS-2 School Age Form (N: 10 TD, 8 ASD). The SRS-2 is a measure designed to capture a wide range of social behaviors that occur across a variety of settings, with an emphasis on skills related to social communication and social reciprocity. The SRS-2 allowed us to examine social impairments that are common to ASD, but occur in TD individuals as well (participant characteristics are presented in Table 1). This measure was included as a way of examining whether the ERP components that differentiate own from other name in TD and ASD children are correlated with social skills that are commonly impaired in ASD.

**Table 1:** Participant characteristics.

	Full TD sample (*n* = 19)	ASD sample (*n* = 13)	Group comparison
**Sex**	13 male, 6 female	11 male, 2 female	χ^2^ = 1.08, p = .30
**Chronological age, in months** Mean (SD)	51.22 (9.05)	52.29 (9.43)	t(30) = 0.32, p = .75
**MSEL verbal t-score** Mean (SD)	53.84 (7.34)	36.08 (10.79)	t(30) = 5.55, p < .001
**MSEL nonverbal t-score** Mean (SD)	55.53 (8.00)	36.04 (12.73)	t(30) = 5.33, p < .001
**SRS-2 total t-score** Mean (SD)	46.61 (7.69)	70.46 (15.69)	t(16.2*) = -5.06, p < .001
**SRS-2 social communication** **t-score** Mean (SD)	46.61 (8.07)	70.38 (15.66)	t(16.5*) = -4.70, p < .001
**SRS-2 social motivation** **t-score** Mean (SD)	48.89 (6.86)	65.85 (14.50)	t(15.9*) = -3.91, p = .001
**Vineland-II Socialization Domain Standard Score** Mean (SD)	103.53 (12.47)	82.73 (12.64)	t(26) = 4.29, p < .001

MSEL = Mullen Scales of Early Learning. SRS-2 = Social Responsiveness Scale-2. Vineland-II = Vineland Adaptive Behavior Scales–II. MSEL verbal t-score = average of receptive and expressive language t-scores. MSEL nonverbal t-score = average of visual reception and fine motor t-scores.

* Because variance in scores was found to differ statistically between groups, a Satterthwaite corrected t-test was employed.

For the ASD group, diagnosis was confirmed using “clinical best estimate” standards: findings from the Autism Diagnostic Observation Schedule–Second Edition (ADOS-2 [32]), Social Communication Questionnaire (SCQ [28]), a follow up clinical SCQ-based developmental interview, the phenotypic data described above, and expert clinical judgment. Diagnoses were based on criteria from the Diagnostic and Statistical Manual of Mental Disorders, Fifth Edition (DSM-5 [33]).

Following developmental and diagnostic testing, participants underwent multiple experimental paradigms, depending on recruitment needs and the participant’s tolerance level. In order to minimize the effects of fatigue and maximize successful data collection, participants with ASD completed the study over the course of two appointments, with developmental and ASD diagnostic assessments occurring on the first day and experimental tasks on the second day. Appointments were separated by no more than three weeks. Since they were not required to complete the ADOS-2, TD participants were scheduled for either one or two days according to participant preference. Four TD children were re-recruited from previous participants to take part in the present experiment. These children had gaps of more than three weeks between developmental testing and EEG data collection. Since chronological age was measured at the date of EEG acquisition, this did not influence the accuracy of group matching. All parents of children who took part in the study reviewed and signed an approved informed consent form. The protocol was approved by CHOP’s Institutional Review Board.

A total of 19 TD participants and 13 participants with ASD provided enough high-quality data to be included in final analyses. Within each diagnostic group, included participants did not significantly differ from excluded participants in chronological age (ASD: t(27) = -0.70, p = .49; TDC: t(28) = -0.67, p = .51), verbal mental age (ASD: t(27) = 0.94, p = .36; TDC: t(28) = -0.48, p = .64), or nonverbal mental age (ASD: t(27) = 0.07, p = .94; TDC: t(28) = -0.33, p = .74). Table 1 presents sample demographics and descriptive statistics of the TD and ASD participants included in both experiments. Means and standard deviations for social variables are also included. On the SRS-2, t-scores from 60–65, 66–75, and 76 or above are considered mildly, moderately, and severely elevated (respectively); t-scores less than 60 are considered to be in the normative range [30]. The Vineland-II Socialization Domain Standard Score is standardized to a mean of 100 and standard deviation of 15.

### Stimuli and paradigm

The experiment included six auditory stimuli designed to elicit social and nonsocial brain responses to familiar and unfamiliar stimuli: 1) child’s own name called by the child’s parent, 2) nonsense name matched in number of syllables and duration to child’s name called by the child’s parent, 3) child’s own name called by an unknown research assistant, 4) the same nonsense name called by an unknown research assistant, 5) familiar music stimuli, 6) unfamiliar music stimuli. Music stimuli were included in the experimental design to function as salient auditory stimuli that might capture brain response to familiar versus unfamiliar nonsocial stimuli. Music stimuli were instrumental, not vocal, auditory stimuli. Their familiarity was based on parent rating of the child’s knowledge of specific music (e.g., theme songs from cartoons or movies).

All parent and stranger stimuli were recorded in a soundproof room, ahead of the experimental session. The nonsense names were determined using Wuggy [34], a freely available pseudoword generator. The nonsense name stimuli had a different first phoneme than the child’s own name that was randomly selected. Nonsense names were chosen based on amplitude to match the pattern of the child’s own name. The voice itself was not manipulated in either condition; both speakers (child’s parent and research assistant, matched on sex) were recorded calling both the child’s own name and the nonsense name. Both speakers were instructed to call the child’s name and the nonsense name as though they were trying to get the child’s attention. Speakers were instructed to call each name nine times, varying the speed of the name-call (three short, three medium, and three long). In order to ensure that the stimuli were closely matched on duration and intonation, the child’s parent was always recorded prior to the research assistant, and the research assistant was asked to emulate the intonation and speed of the child’s parent. This procedure provided nine possible stimuli for each condition of varying lengths.

After recording, stimuli were clipped using the Audacity program [35]. Researchers manually selected the four stimuli (one from each condition) that were most closely matched on duration and listened to the stimuli to verify that the intonation was subjectively comparable. All stimuli were normalized to 70 decibels (dB) in the Praat program [36]. The own name stimuli ranged in duration from 370–1567 ms (mean: 797 ms; SD: 192 ms); the nonsense name stimuli ranged from 461–1584 ms (mean: 790 ms; SD: 190 ms). Although stimuli ranged in duration across participants, prior ERP research suggested that comprehension of spoken words is not reliant on the length of the word [37]. Additional ERP literature found evidence of infants discriminating between their own name and other sounds from the first phoneme of the word [1]. We followed the suggestions of these studies and used speech stimuli of different durations in our experiment. The response to name paradigm was presented via E-Prime 2.0. Software (Psychology Software Tools, Pittsburg, PA, USA). Presentation of the six stimuli was randomly ordered, with equal probability for each stimulus. The experiment consisted of up to four blocks, for a total of 1,056 trials across stimuli, and included intertrial intervals (ITI) that varied randomly between 575, 625, 675, and 725 ms.

### Procedures

Children were tested in a sound-attenuated room. Participants were seated next to a research assistant and in front of a computer monitor. They were told that they would hear some sounds while they watched a silent movie on the screen. Movies were chosen ahead of testing and consisted of cartoons, or abstract shapes and scenes. Children were instructed to stay as still and quiet as possible while the sounds were playing. Stimuli were presented via stereo speakers positioned on either side of the monitor at an average intensity of 70 dB.

### Analysis of ERP data

EEG was collected using a 128-channel Hydrocel Geodesic Sensor Net (Electrical Geodesics Inc., Eugene, Oregon), with channels 125, 126, 127, and 128 removed to enhance net comfort for young children. Data were recorded continuously using NetStation 4.5.7 software with a sample rate of 500 Hz, referenced to electrode Cz. Impedances were kept below 50 kilo-ohms. Following data collection, recordings were highpass filtered (cutoff = 0.1Hz) and notch filtered (notch = 60 Hz) to remove low-frequency and electrical noise, respectively. The data were then segmented to include a 100 ms pre-stimulus baseline and a 1000 ms post-stimulus period.

Repeated timing tests over the course of the study revealed that timing offsets between the stimulus presentation tags in NetStation and actual stimulus presentation exhibited some drift during the experiment, such that offsets decreased systematically and linearly as the experiment progressed. That is, while the timing of the actual stimulus presentation was not changing, the placement of the stimulus offset marker in NetStation was found to be drifting closer to the actual stimulus onset over the course of the experiment. In addition, the rate of drift increased linearly over time, with later experimental administrations showing faster changes in offsets over the course of the experiment. The change in drift over time was remarkably predictable; linear regression demonstrated that date of data collection predicted 84% of variance in offset slope (*p* < .001), with a maximum residual value of .002 ms/s and a mean residual value of 5.21e-20 ms/s. To correct for these timing changes, individual timing tests were run for each participant’s stimuli. Trials in the EEG recording session were binned into 6-minute blocks, as this was found to ensure a maximum offset range of two ms, even with the largest possible amount of intra-experimental offset drift. Offsets within each block were then determined by taking the initial offset for a given stimulus and adjusting based on the linear regression model of offset slope by date of data collection. These adjusted offsets were used to calculate stimulus onset time for segmentation.

Following segmentation, an automatic artifact detection tool was applied to the data, which marked individual channels as bad if their amplitude range exceeded 100 mV and marked trials as bad if they contained more than 12 bad channels (10% of the total number of electrodes). Data were inspected manually by a trained research assistant to identify noisy channels and trials containing artifacts, including eye blinks, gaze shifts, and movement artifacts, using the same threshold of 12 bad channels to mark a trial bad. Participants were excluded from further analysis if they had fewer than 30 good trials in any experimental condition. See Table 2 for the average number of included and excluded trials per participant in each condition in the ASD and TDC groups. Following manual inspection, bad channels were replaced using a spherical spline interpolation algorithm [38]. The trials within each condition were then averaged, re-referenced to an average reference, and baseline corrected for each individual.

**Table 2:** Average number of included and excluded trials per participant in each condition for each diagnostic group.

		TDC	ASD
		Mean (SD)	Mean (SD)
**Parent-Own name**	Included	64 (18)	49 (13)
	Excluded	72 (31)	77 (37)
**Parent-Other name**	Included	62 (17)	48 (12)
	Excluded	74 (30)	79 (38)
**Stranger-Own name**	Included	62 (19)	49 (11)
	Excluded	75 (32)	78 (38)
**Stranger-Other name**	Included	64 (19)	50 (12)
	Excluded	73 (32)	76 (36)

### ERP components

**Fig 1:**
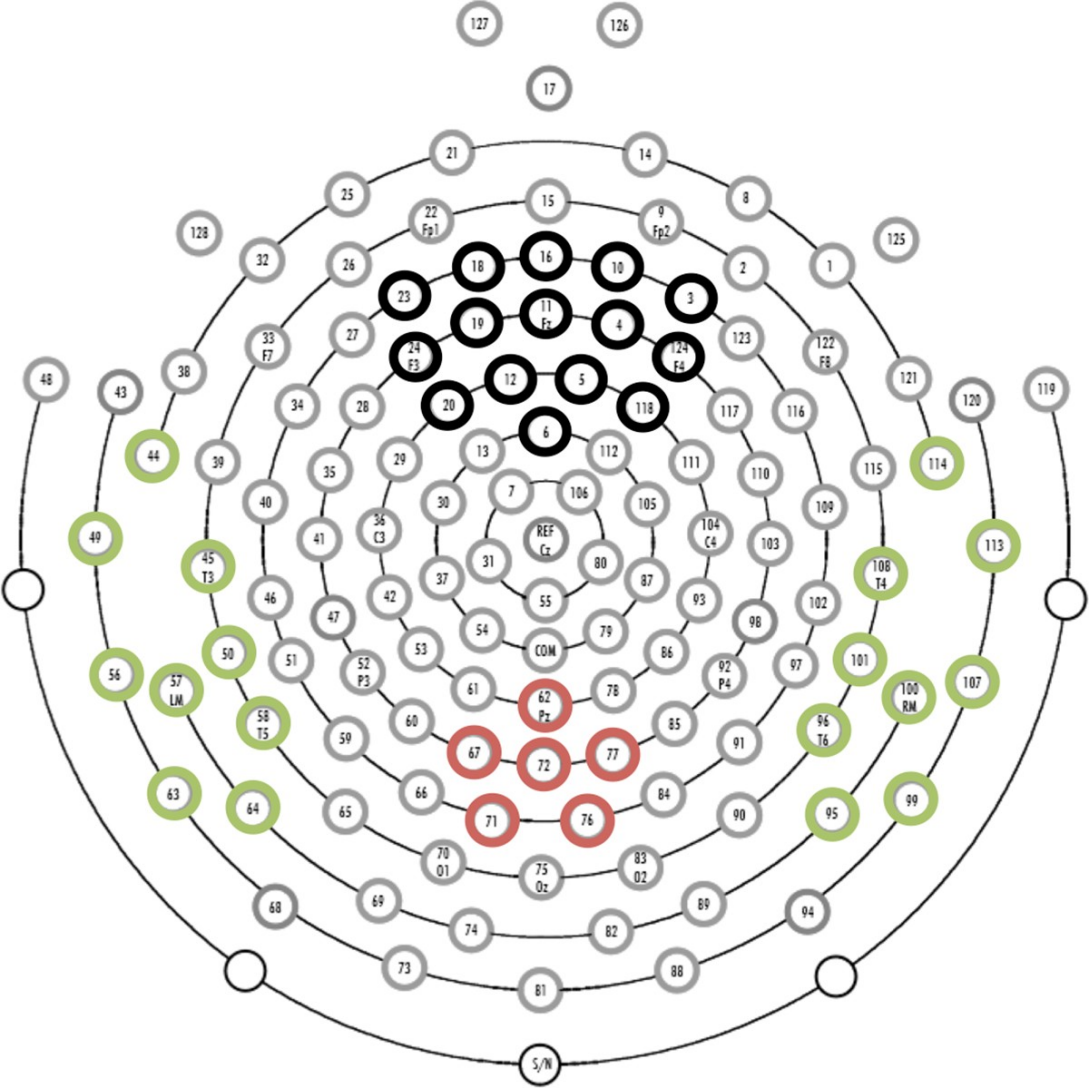
Selected electrodes in frontal (black), temporal (green), and parietal (red) areas.

**Fig 2:**
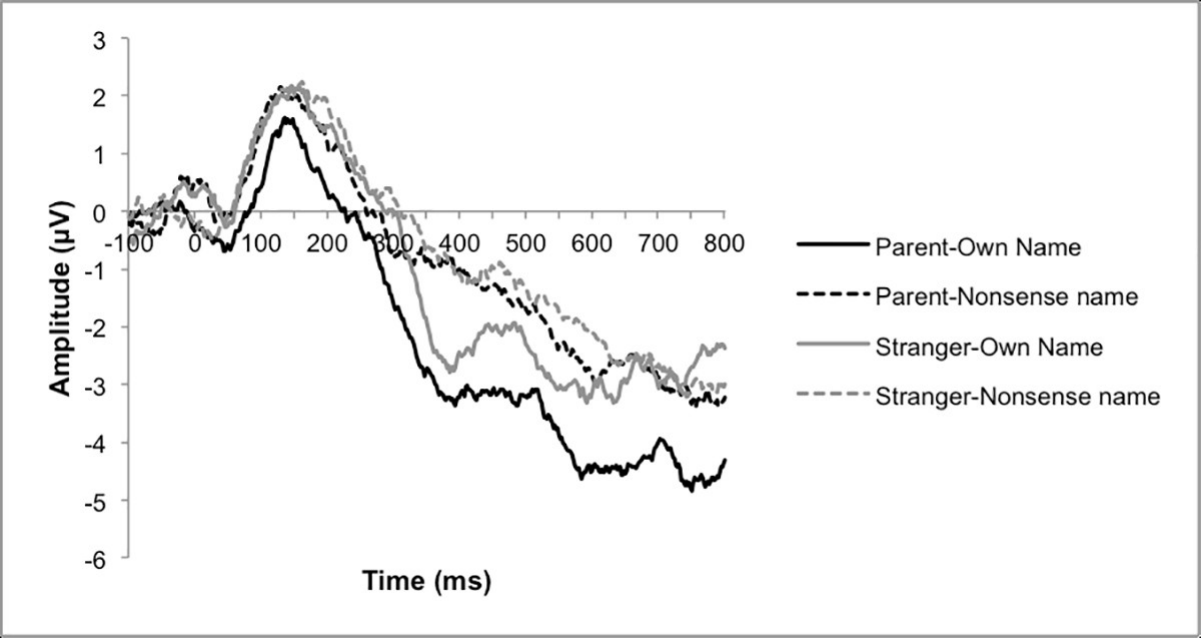
ERP waveforms over frontal regions in the full TD sample in each condition.

Analyses focused on the N100, SON negativity, and LPP components, given the previous findings supporting these components as indices of response to name in typical adults. The P300 was not investigated, as the present study did not utilize an oddball paradigm. Electrode locations and time windows used for analysis of these components were based on prior literature in conjunction with visual inspection of individual and grand averaged waveforms. The N100 was observed over left and right temporal regions (left: channels 44, 45, 49, 50, 56, 57, 58, 63, 64; right: channels 95, 96, 99, 100, 101, 107, 108, 113, 114), consistent with previous studies of speech processing in children in this age range [39]. A SON negativity component was identified over midline frontal sites (channels 3, 4, 5, 6, 10, 11, 12, 16, 18, 19, 20, 23, 24, 118, 124), comparable to its location in older children [7] and typical adults [10]. The LPP was extracted over parietal regions (channels 62, 67, 71, 72, 76, 77), which was again expected based on prior literature [6,8,14,18,19]. See Fig 1 for a visual depiction of selected electrode regions. The following time windows were selected for each component: 50–210 ms for the N100, 550–800 ms for the LPP, and 320–450 ms for SON negativity. While previous studies have identified the SON negativity component occurring prior to 340 ms in adults [8,10], it clearly fell later in the epoch for the children in the present study (Fig 2), so a later time window was used. For the N100 component, which displayed a clear and consistent negative peak across participants, peak amplitude and latency to peak amplitude were extracted. For the SON negativity and LPP components, mean amplitude in the time window was extracted.

Visual inspection revealed a very different pattern of brain response to music stimuli than to speech stimuli. This was particularly true for the later cognitive stage of processing. Thus, the music stimuli did not appear to serve as a comparable nonsocial comparison to speech stimuli as intended. For this reason, the present analysis and discussion focus solely on the speech stimuli in order to elucidate the neural indices of response to name in young children, and leave possible analysis of music stimulus processing for a future report.

### Statistical analysis

2x2 repeated measures ANOVAs were carried out for each component with name (SON vs. nonsense name) and voice (familiar vs. unfamiliar) as within-subjects factors. For the N100 component, hemisphere (left vs. right) was included as an additional within-subjects factor, because it was extracted from lateralized rather than midline regions. In Experiment 2, group (TD vs. ASD) was added as a between-subjects factor. The direction of significant effects was investigated by inspecting estimated marginal means. In order to determine whether results were influenced by the imbalanced (though not statistically significantly different) sex ratios between the two groups, the analyses in Experiment 2 were run once more using a subsample of the TD group (*n* = 16) matched to the ASD group on age (ASD: mean age = 52.29 months; TD: mean age = 51.16 months; *t*(27) = 0.31, *p* = .76) and sex ratio (ASD: 15.4% female; TD: 18.8% female; χ^2^ = 0.06, *p* = .81). This subsample was created by removing three girls from the full TD sample.

Given the importance of differential responding to one’s name to social development, correlations were carried out in Experiment 2 between components that exhibited group differences (main or interaction effects of group) or differential response to one’s own name (main or interaction effects of name) and measures of social skills (SRS-2 total t-score, SRS-2 social communication t-score, SRS-2 motivation t-score, and Vineland-II Socialization Domain Standard score). Correlations were Bonferroni corrected for multiple comparisons.

## Results

### Experiment 1

#### N100

There was a marginally significant main effect of name on peak amplitude of the N100, with more negative responses to nonsense name stimuli than to the subject’s own name (SON) stimuli (*F*(1,18) = 3.92, *p* = .06, *η*_p_^2^ = .179; mean amplitude to SON = -4.16; mean amplitude to nonsense name = -4.77). No significant effect of voice (*F*(1,18) = .46, *p* = .46, *η*_p_^2^ = .03) or hemisphere (*F*(1,18) = 1.90, *p* = .19, *η*_p_^2^ = .10) was found, and there were no significant interaction effects (*p*s ≥ .21). No significant or trending effects were observed for N100 latency (*p*s ≥ .24).

#### SON negativity

The ANOVA revealed a significant main effect of name with more negative mean amplitudes to the subject’s own name (SON) stimuli (*F*(1, 18) = 6.14, *p* = .02, *η*_p_^2^ = .25; mean amplitude to SON = -2.63; mean amplitude to nonsense name = -0.94). There was no significant main effect of voice or significant interaction effect between name and voice. See Fig 2 for grand average waveforms recorded from frontocentral electrodes.

#### LPP

The ANOVA did not reveal any significant main effects of voice (*F*(1,18) = 2.97, *p* = .10, *η*_p_^2^ = .14) or name (*F*(1,18) = .07, *p* = .80, *η*_p_^2^ = .004) in the late parietal positivity (LPP) component. Additionally, no significant interaction effect was found (*p* = .89).

### Experiment 2

#### N100

There was a significant main effect of hemisphere on peak amplitude of the N100, with more negative responses in the left hemisphere than the right hemisphere (*F*(1, 30) = 5.26, *p* = .03, *η*_p_^2^ = .15; mean amplitude in the left hemisphere = -4.99; mean amplitude in the right hemisphere = -4.27). No significant effect of name (*F*(1, 30) = 1.56, *p* = .22, *η*_p_^2^ = .05), voice (*F*(1, 30) = .07, *p* = .80, *η*_p_^2^ = .002), or group (*F*(1, 30) = .61, *p* = .44, *η*_p_^2^ = .02) was found, and there were no significant interaction effects (*p*s ≥ .11). A marginally significant main effect of hemisphere was observed for N100 latency, in which participants exhibited faster responses in the right hemisphere than they did in the left, on average (*F*(1, 30) = 3.64, *p* = .07, *η*_p_^2^ = .11; mean latency in the left hemisphere = 134.96 milliseconds; mean latency in the right hemisphere = 129.77 milliseconds). No significant effect of name (F(1, 30) = 1.25, p = .27, *η*_p_^2^ = .04), voice (F(1, 30) = 1.74, p = .20, ηp2 = .06), or group (F(1, 30) = 2.27, p = .14, *η*_p_^2^ = .07) was found, and there were no significant interaction effects (ps ≥ .15). The pattern of results did not change when analyses were conducted using a sex-matched subsample of the TD group, with the exception of the main effect of hemisphere on N100 latency, which became statistically significant (F(1, 27) = 5.00, p = .03, *η*_p_^2^ = .16).

#### SON negativity

**Fig 3:**
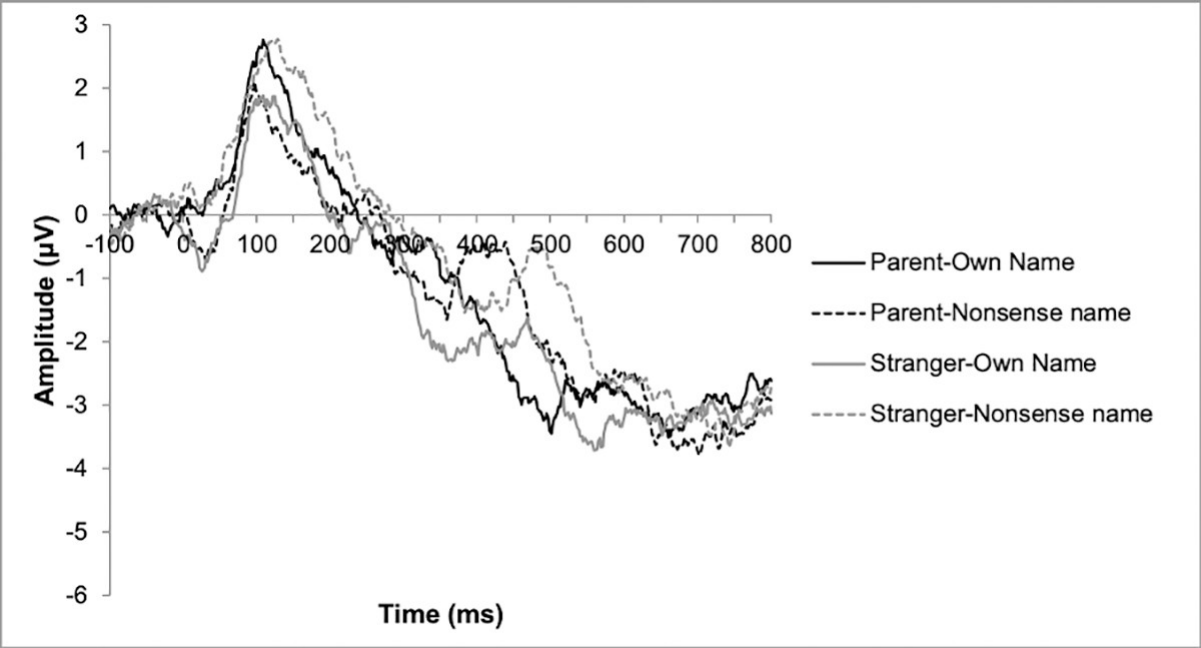
ERP waveforms over frontal regions in the ASD sample in each condition.

The ANOVA revealed a significant main effect of name, such that SON elicited a more negative mean amplitude than did the nonsense name across both groups (*F*(1, 30) = 7.12, *p* = .01, *η*_p_^2^ = .19; mean amplitude to SON = -2.19; mean amplitude to nonsense name = -0.97). No main effect of voice (*F*(1, 30) = .001, *p* = .98, *η*_p_^2^ < .001) or group (*F*(1, 30) = .43, *p* = .52, *η*_p_^2^ = .01) was found, and no significant interaction effects were found (*p*s ≥ .17). See Fig 3 for ERP waveforms recorded over frontal regions in the ASD group. The pattern of results did not change when analyses were conducted using a sex-matched subsample of the TD group, and the main effect of name remained significant (*F*(1, 27) = 8.61, *p* = .007, *η*_p_^2^ = .24).

#### LPP

As in Experiment 1, the ANOVA did not reveal any significant main effects of voice (*F*(1,30) = 2.40, *p* = .13, *η*_p_^2^ = .07), name (*F*(1,30) = .19, *p* = .67, *η*_p_^2^ = .01), or group (*F*(1,30) = .31, *p* = .58, *η*_p_^2^ = .01) in the late parietal positivity (LPP) component. Additionally, no significant interaction effects were found (*p*s ≥ .45). The pattern of results was the same in the analyses using the sex-matched subsample of the TD group.

#### Correlations with social variables

Because of the significant effect of name for the SON negativity component, bivariate correlations were carried out between the mean amplitude of the SON negativity component in both the familiar and unfamiliar voice condition and the SRS-2 total t-score, SRS-2 social communication t-score, SRS-2 motivation t-score, and Vineland-II socialization domain standard score. Correlations were carried out separately for the TD and ASD groups. In the ASD group, the mean SON negativity amplitude to an unfamiliar voice correlated significantly with SRS-2 social motivation t-score (*r* = .60, *p* = .03; *n* = 13) and Vineland-II socialization score (*r* = -.63, *p* = .04, *n* = 11), such that greater SON negativity was associated with better social skills. However, these correlations were not significant after stringent Bonferroni correction for multiple comparisons. SON negativity to an unfamiliar voice was not correlated significantly with SRS-2 social communication t-score (*r* = .36, *p* = .23, *n* = 13) or total t-score (*r* = .42, *p* = .15, *n* = 13), and SON negativity to a familiar voice was not correlated significantly with any social variables (SRS-2 motivation: *r* = -.05, *p* = .87, *n* = 13; SRS-2 social communication: *r* = .08, *p* = .80, *n* = 13; SRS-2 total: *r* = .06, *p* = .85, *n* = 13; Vineland-II socialization: *r* = -.35, *p* = .29, *n* = 11) in the ASD group. No correlations emerged as significant in the TD group, either with SON negativity to a familiar voice (SRS-2 motivation: *r* = -.10, *p* = .70, *n* = 18; SRS-2 social communication: *r* = .05, *p* = .84, *n* = 18; SRS-2 total: *r* = .10, *p* = .70, *n* = 18; Vineland-II socialization: *r* = .01, *p* = .98, *n* = 17) or with SON negativity to an unfamiliar voice (SRS-2 motivation: *r* = -.05, *p* = .85, *n* = 18; SRS-2 social communication: *r* = .12, *p* = .65, *n* = 18; SRS-2 total: *r* = .21, *p* = .41, *n* = 18; Vineland-II socialization: *r* = .05, *p* = .86, *n* = 17).

## Discussion

The aim of this study was to examine the neural mechanisms underlying the auditory processing response to name in typically developing preschoolers, with an additional exploratory pilot comparison of a sample of chronological age-matched children with ASD. This is the first study to 1) examine these mechanisms using ERPs in preschool-aged children, and 2) investigate the differential contribution of name (own vs. other) and speaker familiarity (parent vs. stranger). These data present novel information on the factors that influence how naturalistic, socially salient auditory information is processed in young children.

Although previous research has established the SON negativity component in adults and older children, the current report is the first to demonstrate the presence and function of this component in TD preschoolers as young as three years old. These data are some of the first to provide brain-based evidence that typically developing children orient their attention to and selectively process their own name regardless of the speaker; the amplitude of the SON negativity component was not influenced by who called the child’s name. That this component was present despite the equal probability of all speech stimuli provides brain-based evidence of the social importance of one’s own name regardless of the speaker or how often the name was repeated.

In addition, although our data provide evidence of the SON negativity in children, the timing of the component (320-450ms) was later than the adult ERP literature has indicated (170-270ms [10]). Similarly, a previous study of ERP response to name in school-age children found that the SON negativity component was delayed relative to adults (280-320ms), though not as delayed as in the present study [7]. These findings suggest that the neural systems underlying this component are present in preschool-aged children but continue to mature throughout childhood. The age at which this component reaches adult-like speed remains unknown.

We did not find evidence of the LPP in typically developing children between three and five years of age. Previous research has demonstrated that one of the cerebral sources of the LPP response to one’s own name is the right temporo-parietal junction (rTPJ; Nijhof et al., 2018), a brain region that has been shown to support the ability to make inferences about the mental states of others (known as “theory of mind”; Saxe & Wexler, 2005). More specifically, the rTPJ is thought to contribute to more abstract components of theory of mind, including the ability to understand and attribute beliefs to others, a skill which is not thought to develop until the age of four (Saxe & Powell, 2006). It is possible that the increased LPP response to one’s own name indicates that individuals tend to engage in more automatic reasoning about the mental states of others (e.g., “Why might that person be calling my name?”) when they hear someone calling their name than when they hear someone calling another name. If this is the case, then the lack of LPP response in the present study may simply reflect the fact that such theory of mind processes are absent or still emerging in children in this age range. However, the LPP has only been observed in adults and research has yet to establish a developmental timeline of this component in childhood. Future research should examine how and when this component develops in childhood and whether it is tied to theory of mind ability.

A marginally significant main effect of name in the N100 provides provisional evidence that own and nonsense names are processed differently from the earliest stages of auditory processing. Prior research on SON processing in TD adults has posited that the N100 may serve as a signal of automatic attentional orienting [5]. Our results indicated a greater N100 response to nonsense name than own name in TD children, regardless of the speaker, suggesting that TD children display greater early automatic orienting to nonsense names than their own name. This contradicts prior research on this component in adults [5] and young children [7], which found greater responses to SON. However, Bathelt et al. [7] used real names (not pseudo-names) as control stimuli and included an additional word (“Hey”) before their SON and unfamiliar name stimuli to indicate communicative intent. It is possible that the differences between our findings and Bathelt and colleagues’ findings are related to the novelty of the control stimuli and/or to differing degrees of social-communicative context. For instance, it could be the case that TD children are less likely to automatically orient to their own name in a context in which communicative intent is less clear, and that the nonsense names employed in the present study were more novel and attention-grabbing than the unfamiliar names used in previous studies. Furthermore, previous research has examined the N100 component using central electrodes [5], while our study examined this component in the temporal regions, in line with research on speech processing in similar-aged young children [39]. All of these factors could have contributed to differences in N100 findings in the present study.

In addition, our analyses including both TD and ASD children revealed a lateralization of early auditory responses in the N100 component, with significantly greater amplitude in the left hemisphere and marginally significantly faster latency in the right hemisphere. The N100 amplitude results are consistent with the well-known left lateralization of phonological processing in both children and adults (see [40] for a review). However, the marginally significant finding of slower N100 latency in the left hemisphere was more unexpected. Given the left-hemisphere bias for language processing, it is possible that slower N100 latency is a reflection of deeper processing in the left hemisphere. Alternatively, given the marginal significance and somewhat small size of this effect (*η*_*p*_^*2*^ = .11), this finding may simply be a result of the relatively small sample included in the present study. To help resolve these questions, we hope to examine N100 latency and amplitude in future research with larger sample sizes of children in this same age range.

The current results also provide preliminary evidence of selective processing of SON in both TD children and children with ASD. The main effect of name on the SON negativity component was significant across groups, revealing a pattern of stronger brain response to own name than to nonsense name across groups. The absence of a significant group interaction effect suggests that both TD and ASD children exhibited a SON negativity response to their own name to the same approximate degree. These findings indicate that both TD children and children with ASD differentiate their own name from other sounds.

Furthermore, the amplitude of SON negativity to an unfamiliar speaker correlated with measures of social motivation and social skills in the ASD group. The significance of these correlations did not survive correction for multiple comparisons, which is not surprising given the small sample size. However, in light of the large effect sizes of these correlations (|*r*| ≥ .6), further consideration is warranted. If replicated, these correlations might suggest that individuals who exhibit stronger neural response to their own name also exhibit higher social motivation and better social skills. The fact that such a relationship was not observed in the TD group may be a result of lower variability in social skills in the TD group; indeed, there was significantly less variability in social motivation scores in the TD than ASD group (*p* = .005), but not in social skills (*p* = .93). Alternatively, it may indicate that TD children are better able to compensate for attenuated response to name than children with ASD, resulting in fewer negative consequences of early differences in response to name for TD children. Notably, SON response to a *familiar* voice did not show similar correlations with social skills in the ASD group. This could be due to the fact that the SON negativity to a familiar voice appeared to peak outside the component’s time window (Fig 3), potentially attenuating (and thus restricting the range of) the estimate of the amplitude of the SON negativity response to a familiar voice in the ASD group. Another reasonable explanation is that orienting to an unfamiliar voice is less automatic and more reflective of social motivation than orienting to a parent’s voice for children with ASD. Given the small sample size included in this experiment, the degree to which these results are driven by reliable signal rather than noise remains uncertain. Future research with larger samples can clarify the nature of the differences in brain response to SON in children with ASD and how they relate to social and communicative impairment in this population.

### Limitations and future directions

A primary limitation of the current study was the small sample size, particularly for the ASD group described in Experiment 2. Those results are presented as an exploratory pilot investigation designed to promote and guide future investigation on this important topic. Thus, we presented marginally significant results in both Experiments 1 and 2. Future research examining indices of response to name in children should examine whether these initial findings of the current study replicate in larger samples of both typically developing children and children with ASD.

In the current study we found that while certain indices of response to name previously reported in typical adults are present by three years of age, the brain response to one’s own name may be still maturing. In particular, the SON negativity component identified in preschoolers occurred later than in typical adults, and the LPP component was entirely absent. Future longitudinal research is needed to fully characterize the emergence and maturation of these components over the course of early development. These studies should also investigate the relationships between brain response to one’s own name and subsequent social and linguistic development. Such findings could hold implications for our understanding and prediction of both typical and atypical development.

In addition, the present study matched the TD and ASD samples based on chronological age only. This was done to control for length of own-name exposure, based on the assumption that brain response to one’s own name would be more closely linked with experience than with developmental level. However, response to name may also be associated with general maturation or socio-communicative factors. As a result, it is not certain whether the differences in brain response observed in the ASD group in the present study are attributable to diagnostic status, developmental level, or a combination of the two. Future research incorporating control groups matched on developmental age or social skills would help to elucidate the particular causes of the distinct neural response to SON observed in children with ASD.

In order to control for the familiarity of the non-SON speech stimuli, we utilized non-semantically meaningful nonsense words. Although we ensured that all name stimuli (SON and nonsense name) were called with the same intonation to mimic name calls, it is possible that our SON negativity finding is capturing responses to special or meaningful words, instead of response to name. However, our results parallel those of studies which have used real names as control stimuli in adult ERP studies. Future research should more closely examine the specificity of the SON negativity component in response to name in children compared to response to preferred/meaningful words, such as a family member’s name or the name of a favorite toy.

## Conclusions

The current findings are the first to indicate that the SON negativity component exists in typically developing three- to five-year-old children, albeit in a developmentally appropriate later time window than in typically developing adults. Furthermore, our preliminary findings with a chronological age-matched sample of children with ASD suggest that they also exhibit the SON negativity component. Future research should more specifically examine how and when this component develops in both populations, as this brain response likely contributes not only to social development but also to linguistic and cognitive development as children learn to use their names to guide and orient their attention and establish relevant environmental cues.
